# Supplementary material for: A systematic review and meta-analyses on initiation, adherence and outcomes of antiretroviral therapy in incarcerated people
Source: PLoS One. 2020 May 18;15(5):e0233355. doi: 10.1371/journal.pone.0233355 (PMC7233580; doi:10.1371/journal.pone.0233355)
Supplement: S2 Table — Data extraction form adapted from Cochrane review format for data extraction. (DOCX) [file pone.0233355.s002.docx]

**Data extraction form**

| Author (Year) | Country | Population | Study Design | Measurement | Types of exposures | Findings | Conclusions |
| --- | --- | --- | --- | --- | --- | --- | --- |
|  |  |  |  |  |  |  |  |
|  |  |  |  |  |  |  |  |
