# Supplementary material for: A systematic review and meta-analyses on initiation, adherence and outcomes of antiretroviral therapy in incarcerated people
Source: PLoS One. 2020 May 18;15(5):e0233355. doi: 10.1371/journal.pone.0233355 (PMC7233580; doi:10.1371/journal.pone.0233355)
Supplement: S3 Table — Quality assessment results for quantitative studies included in the final review using EPHPP Tool. (DOCX) [file pone.0233355.s003.docx]

**Quality assessment results for quantitative studies included in the final review using EPHPP Tool**

| Article | Selection Bias | Study Design | Confounders | Data Collection Methods | Withdrawals and Drop-Outs | Global Rating |
| --- | --- | --- | --- | --- | --- | --- |
| 1. ***Linkage to care/ART Initiation Studies*** | | | | | | |
| Lucas et al (2016) | +/- | - | - | - | x | - |
| Mostashari et al (1998) | +/- | - | - | - | x | - |
| Monarca et al (2015) | +/- | - | - | - | x | - |
| White et al (2001) | +/- | - | +/- | - | x | - |
| Bick et al (2016) | - | - | - | - | x | - |
| Sgarbi et al (2015) | +/- | - | - | - | +/- | - |
| Makombe et al (2007) | +/- | +/- | - | - | +/- | - |
| Culbert et al (2016) | +/- | - | + | + | x | +/- |
| Jaffer et al (2012) | +/- | - | - | - | x | - |
| Pérez-Molina et al (2002) | +/- | - | +/- | - | x | - |
| Seth et al (2015) | +/- | - | - | - | x | - |
| Altice et al (2001) | +/- | - | + | +/- | x | +/- |
| 1. ***ART adherence Studies*** | | | | | | |
| Soto Blanco et al^a^ (2005) | +/- | - | +/- | +/- | x | +/- |
| Milloy et al (2011) | - | +/- | + | + | x | +/- |
| Soto Blanco et al^b^ (2005) | +/- | - | +/- | +/- | x | +/- |
| White et al (2006) | - | - | - | + | + | - |
| Palepu et al (2004) | + | +/- | + | + | x | + |
| Paparizos et al (2013) | - | - | - | - | x | - |
| Ines et al (2008) | +/- | - | - | +/- | x | - |
| Subramanian et al (2016) | - | - | - | +/- | x | - |
| 1. ***ART outcomes studies*** | | | | | | |
| Davies and Karstaedt (2012) | +/- | +/- | - | - | x | - |
| Eastment et al (2017) | - | - | - | - | x | - |
| Meyer et al (2015) | +/- | +/- | + | - | x | +/- |
| Meyer et al (2014) | +/- | +/- | + | +/- | x | + |
| Mpawa et al (2017) | +/- | - | +/- | - | x | - |
| Nasrullah et al (2016) | + | - | + | - | x | - |
| Palepu et al (2003) | - | +/- | +/- | - | x | - |
| Stephenson et al (2005) | +/- | +/- | +/- | - | x | +/- |
| Springer et al (2004) | +/- | +/- | - | - | x | - |
| Chan et al (2015) | - | - | - | - | x | - |
| dos Santos Bet et al (2018) | +/- | +/- | +/- | - | - | - |
| Westergaard et al (2011) | - | +/- | + | +/- | - | - |
| Telisinghe et al (2016) | + | +/- | - | - | x | - |
| Meyer et al (2014) | +/- | +/- | +/- | - | x | +/- |

**+** Strong; **+/-** Moderate; **-** Weak; **x** Not applicable

Global rating: **Strong:** No weak rating; **Moderate:** one weak rating; **Weak**: two or more weak ratings
