# Supplementary material for: A systematic review and meta-analyses on initiation, adherence and outcomes of antiretroviral therapy in incarcerated people
Source: PLoS One. 2020 May 18;15(5):e0233355. doi: 10.1371/journal.pone.0233355 (PMC7233580; doi:10.1371/journal.pone.0233355)
Supplement: S1 File — A review protocol registered in international prospective register of systematic reviews (PROSPERO). (PDF) [file pone.0233355.s004.pdf]

National Institute for Health Research

University of York
Centre for Reviews and Dissemination

Systematic review

1. * Review title.

Give the working title of the review, for example the one used for obtaining funding. Ideally the title should state succinctly the interventions or exposures being reviewed and the associated health or social problems. Where appropriate, the title should use the PI(E)COS structure to contain information on the Participants, Intervention (or Exposure) and Comparison groups, the Outcomes to be measured and Study designs to be included.

Systematic review and meta-analysis of initiation, adherence and outcomes of antiretroviral therapy among incarcerated people

2. Original language title.

For reviews in languages other than English, this field should be used to enter the title in the language of the review. This will be displayed together with the English language title.

3. * Anticipated or actual start date.

Give the date when the systematic review commenced, or is expected to commence.

01/02/2019

4. * Anticipated completion date.

Give the date by which the review is expected to be completed.

30/09/2019

5. * Stage of review at time of this submission.

Indicate the stage of progress of the review by ticking the relevant Started and Completed boxes. Additional information may be added in the free text box provided.
Please note: Reviews that have progressed beyond the point of completing data extraction at the time of initial registration are not eligible for inclusion in PROSPERO. Should evidence of incorrect status and/or completion date being supplied at the time of submission come to light, the content of the PROSPERO record will be removed leaving only the title and named contact details and a statement that inaccuracies in the stage of the review date had been identified.
This field should be updated when any amendments are made to a published record and on completion and publication of the review. If this field was pre-populated from the initial screening questions then you are not able to edit it until the record is published.

The review has not yet started: No

Review stage	Started	Completed
Preliminary searches	Yes	Yes
Piloting of the study selection process	Yes	No
Formal screening of search results against eligibility criteria	No	No
Data extraction	No	No
Risk of bias (quality) assessment	No	No
Data analysis	No	No

Provide any other relevant information about the stage of the review here (e.g. Funded proposal, protocol not yet finalised).

6. * Named contact.

The named contact acts as the guarantor for the accuracy of the information presented in the register record.

Terefe Fuge

Email salutation (e.g. "Dr Smith" or "Joanne") for correspondence:

Mr Fuge

7. * Named contact email.

Give the electronic mail address of the named contact.

fuge0002@flinders.edu.au

8. Named contact address

Give the full postal address for the named contact.

Flinders University, College of Medicine and Public Health, GPO Box 2100 | Adelaide SA 5001

9. Named contact phone number.

Give the telephone number for the named contact, including international dialling code.

+61(0)872218445

10. * Organisational affiliation of the review.

Full title of the organisational affiliations for this review and website address if available. This field may be completed as 'None' if the review is not affiliated to any organisation.

Flinders University

Organisation web address:

http://www.flinders.edu.au

11. * Review team members and their organisational affiliations.

Give the title, first name, last name and the organisational affiliations of each member of the review team. Affiliation refers to groups or organisations to which review team members belong.

Mr Terefe Fuge. Flinders University
Dr Emma Miller. Flinders University
Dr George Tsourtos. Flinders University

12. * Funding sources/sponsors.

Give details of the individuals, organizations, groups or other legal entities who take responsibility for initiating, managing, sponsoring and/or financing the review. Include any unique identification numbers assigned to the review by the individuals or bodies listed.

Flinders University

13. * Conflicts of interest.

List any conditions that could lead to actual or perceived undue influence on judgements concerning the main topic investigated in the review.

None

The authors declare that they have no known conflicts of interest.

14. Collaborators.

Give the name and affiliation of any individuals or organisations who are working on the review but who are not listed as review team members.

15. * Review question.

State the question(s) to be addressed by the review, clearly and precisely. Review questions may be specific or broad. It may be appropriate to break very broad questions down into a series of related more specific questions. Questions may be framed or refined using PI(E)COS where relevant.

How does incarceration impact on initiation, adherence and outcomes of antiretroviral therapy?

16. * Searches.

State the sources that will be searched. Give the search dates, and any restrictions (e.g. language or publication period). Do NOT enter the full search strategy (it may be provided as a link or attachment.)

Systematic searches will be carried out on the following databases; Emcare, MEDLINE, PubMed, Scopus, Web of Science, CINAHL and Cochrane Library. The concepts HIV/AIDS, ART and Incarceration will be used to construct the search strategy. The search strategy uses only terms related to exposure (incarceration) and outcomes. The terms will be combined with MEDLINE filter for the concepts under search. The search terms will be adapted for use with other bibliographic databases in combination with database-specific filters for the concepts, where these are available. The search strategy is developed with the help of a qualified librarian. While no restriction will be made in terms of geographical region and year of publication, studies should be published in English language and indexed up to 26 October 2018 to be included in the review. The searches will be re-run just before the final analyses and further studies retrieved for inclusion.

The search strategy for MEDLINE is; HIV or AIDS or HIV-AIDS or Acquired Immunodeficiency Syndrome or

Human immunodeficiency virus AND antiretroviral* or anti-retroviral* or HAART or ART or anti-hiv AND prison* or incarcerate* or imprison* or inmate* or jail* or detention* or "correctional facilities" or "correctional setting" or "house of correction" or custody or convict or detainee.

17. URL to search strategy.

Give a link to a published pdf/word document detailing either the search strategy or an example of a search strategy for a specific database if available (including the keywords that will be used in the search strategies), or upload your search strategy.Do NOT provide links to your search results.

Alternatively, upload your search strategy to CRD in pdf format. Please note that by doing so you are consenting to the file being made publicly accessible.

Do not make this file publicly available until the review is complete

18. * Condition or domain being studied.

Give a short description of the disease, condition or healthcare domain being studied. This could include health and wellbeing outcomes.

Antiretroviral therapy in incarcerated people

19. * Participants/population.

Give summary criteria for the participants or populations being studied by the review. The preferred format includes details of both inclusion and exclusion criteria.

Studies evaluating HIV care during incarceration or the effect of history of incarceration on ART initiation, adherence and outcomes will be considered for review. Those studies focusing only on HIV care use before incarceration or community care linkage after release will be excluded. Studies will be included in the review if they investigated HIV care utilisation before, during and after incarceration comparatively. Studies conducted on specific populations such as certain ethnic groups or population with particular characteristics (e.g. transgender people, men who have sex with men) will be excluded.

20. * Intervention(s), exposure(s).

Give full and clear descriptions or definitions of the nature of the interventions or the exposures to be reviewed.

Articles exploring structural, social and individual level determinants of HIV care utilisation among prisoners will be reviewed. More specifically, studies analysing factors related to access and availability of HIV care; psychosocial factors such as depression, social support, disclosure, stigma and privacy; behavioural factors such as attitude towards ART; health and medication related factors including comorbidity, immunological or clinical status; incarceration related factors such as number and length of imprisonment; and socioeconomic factors including age, sex, and other characteristics will be assessed.

21. * Comparator(s)/control.

Where relevant, give details of the alternatives against which the main subject/topic of the review will be compared (e.g. another intervention or a non-exposed control group). The preferred format includes details of both inclusion and exclusion criteria.

While no restriction will be made based on whether a study has used comparators, non-incarcerated people will be considered as a control group when comparisons are made. Comparator 'Not applicable' for qualitative studies.

22. * Types of study to be included.

Give details of the types of study (study designs) eligible for inclusion in the review. If there are no restrictions on the types of study design eligible for inclusion, or certain study types are excluded, this should be stated. The preferred format includes details of both inclusion and exclusion criteria.

Studies investigating one or more of the following HIV care cascade elements in incarcerated populations will be included in the review; linkage to HIV care, initiation of ART, adherence to ART and outcomes of ART. Both quantitative and qualitative studies will be reviewed without restriction based on type of study design and publication date.

23. Context.

Give summary details of the setting and other relevant characteristics which help define the inclusion or exclusion criteria.

Global studies focusing on one or more of the major components of HIV care cascade (i.e. ART initiation, adherence or outcomes) in the prison population will be included.

24. * Main outcome(s).

Give the pre-specified main (most important) outcomes of the review, including details of how the outcome is defined and measured and when these measurement are made, if these are part of the review inclusion criteria.

Studies reporting one or more of the following outcomes will be included in the review; linkage to HIV care, initiation of ART, adherence to and outcomes of ART in terms of change in CD4 count and viral suppression. No restriction will be made based on the definition of the outcomes. For qualitative studies, description of barriers and facilitators of HIV care utilisation among inmates.

Timing and effect measures

Delay in linkage to HIV care or ART initiation measured using different methods such as WHO clinical staging, CD4 count and determination of time between HIV diagnosis and linkage to care/ART initiation. Adherence to dose at varying period of time (days, weeks and months) measured using different methods (self-report, pharmacy refill, pill count, electronic monitoring cups) at different thresholds. Outcomes of ART measured in terms of CD4 count and viral load at different cut off values.

25. * Additional outcome(s).

List the pre-specified additional outcomes of the review, with a similar level of detail to that required for main outcomes. Where there are no additional outcomes please state 'None' or 'Not applicable' as appropriate to the review

None

Timing and effect measures

Not applicable.

26. * Data extraction (selection and coding).

Describe how studies will be selected for inclusion. State what data will be extracted or obtained. State how this will be done and recorded.

Articles obtained from database searches will be screened for relevance with their titles and abstracts. After removal of duplicate and irrelevant articles, full text review will be performed on the retrieved articles. Corresponding authors of primary studies will be contacted for any missing or unclear information. Data will be extracted using a format adapted from Cochrane Systematic Review Checklist for Data Collection. Separate data extraction formats will be used for treatment initiation, adherence and outcomes categories. Information in the data extraction form include author, year, geographical location, population, method, measurements, exposures, results and outcomes.

27. * Risk of bias (quality) assessment.

Describe the method of assessing risk of bias or quality assessment. State which characteristics of the studies will be assessed and any formal risk of bias tools that will be used.

Two review authors will independently perform quality (risk of bias) assessment of the retrieved articles. The quality assessment will be done using EPHPP Quality Assessment Tool for Quantitative Studies by considering the following characteristics; representativeness of participants (selection bias), study design, control of potential confounders, validity and reliability of data collection methods and completeness of outcome data (withdrawals and dropouts). The criterion of “blindness” will not be considered due to observational nature of most studies in the field. Disagreements between the review authors over the risk of bias in particular studies will be resolved by discussion, with involvement of a third review author where necessary. Risk of bias assessment ‘Not applicable’ for qualitative studies.

28. * Strategy for data synthesis.

Provide details of the planned synthesis including a rationale for the methods selected. This **must not be generic text** but should be **specific to your review** and describe how the proposed analysis will be applied to your data.

We will provide narrative synthesis of the findings across studies in terms of exposures and outcomes. Due to variety of outcomes measured and difference in definition of each of the outcome across studies, we anticipate limited scope for meta-analysis. However, whenever studies have measured the same outcome and exposure with the same definition, we will pool the outcomes using a random-effects meta-analysis, with standardised mean differences for continuous outcomes and risk ratios for binary outcomes, and calculate 95% confidence intervals and two sided P values for each outcome. Standard deviations will be adjusted for studies with small sample size and those in which clustering effects have not been considered. Heterogeneity between studies in effect measures will be determined using Chi^2 test and I^2 statistic. I^2

greater than 50% will be considered as substantial heterogeneity. We will perform sensitivity analysis based on study quality, and subgroup analysis to explore heterogeneity in effect estimate based on study quality and type of exposure. Publication bias will also be detected using a funnel plot.

29. * Analysis of subgroups or subsets.

State any planned investigation of 'subgroups'. Be clear and specific about which type of study or participant will be included in each group or covariate investigated. State the planned analytic approach.

If the necessary data are available, subgroup analysis will be performed for different settings (low and high income countries), types of study designs, measurements and thresholds. Within each study outcome and overall, we will also perform subgroup analysis by age and number of incarceration episodes.

30. * Type and method of review.

Select the type of review and the review method from the lists below. Select the health area(s) of interest for your review.

Type of review

Cost effectiveness
No

Diagnostic
No

Epidemiologic
No

Individual patient data (IPD) meta-analysis
No

Intervention
No

Meta-analysis
Yes

Methodology
No

Narrative synthesis
No

Network meta-analysis
No

Pre-clinical
No

Prevention
No

Prognostic
No

Prospective meta-analysis (PMA)
No

Review of reviews
No

Service delivery
No

Synthesis of qualitative studies
No

Systematic review
Yes

Other
No

Health area of the review

Alcohol/substance misuse/abuse
No

Blood and immune system
No

Cancer
No

Cardiovascular
No

Care of the elderly
No

Child health
No

Complementary therapies
No

Crime and justice
No

Dental
No

Digestive system
No

Ear, nose and throat
No

Education
No

Endocrine and metabolic disorders
No

Eye disorders
No

General interest
No

Genetics
No

Health inequalities/health equity
No

Infections and infestations
No

International development
No

Mental health and behavioural conditions
No

Musculoskeletal
No

Neurological
No

Nursing

No

Obstetrics and gynaecology
No

Oral health
No

Palliative care
No

Perioperative care
No

Physiotherapy
No

Pregnancy and childbirth
No

Public health (including social determinants of health)
Yes

Rehabilitation
No

Respiratory disorders
No

Service delivery
No

Skin disorders
No

Social care
No

Surgery
No

Tropical Medicine
No

Urological
No

Wounds, injuries and accidents
No

Violence and abuse
No

31. Language.

Select each language individually to add it to the list below, use the bin icon to remove any added in error.
English

There is not an English language summary

32. Country.

Select the country in which the review is being carried out from the drop down list. For multi-national collaborations select all the countries involved.

Australia

33. Other registration details.

Give the name of any organisation where the systematic review title or protocol is registered (such as with

The Campbell Collaboration, or The Joanna Briggs Institute) together with any unique identification number assigned. (N.B. Registration details for Cochrane protocols will be automatically entered). If extracted data will be stored and made available through a repository such as the Systematic Review Data Repository (SRDR), details and a link should be included here. If none, leave blank.

34. Reference and/or URL for published protocol.

Give the citation and link for the published protocol, if there is one

Give the link to the published protocol.

Alternatively, upload your published protocol to CRD in pdf format. Please note that by doing so you are consenting to the file being made publicly accessible.

No I do not make this file publicly available until the review is complete

Please note that the information required in the PROSPERO registration form must be completed in full even if access to a protocol is given.

35. Dissemination plans.

Give brief details of plans for communicating essential messages from the review to the appropriate audiences.

Do you intend to publish the review on completion?

Yes

36. Keywords.

Give words or phrases that best describe the review. Separate keywords with a semicolon or new line. Keywords will help users find the review in the Register (the words do not appear in the public record but are included in searches). Be as specific and precise as possible. Avoid acronyms and abbreviations unless these are in wide use.

Systematic review; meta-analysis; antiretroviral therapy; initiation; adherence; outcomes; incarceration

37. Details of any existing review of the same topic by the same authors.

Give details of earlier versions of the systematic review if an update of an existing review is being registered, including full bibliographic reference if possible.

38. * Current review status.

Review status should be updated when the review is completed and when it is published. For newregistrations the review must be Ongoing.
Please provide anticipated publication date

Review_Ongoing

39. Any additional information.

Provide any other information the review team feel is relevant to the registration of the review.

40. Details of final report/publication(s).

This field should be left empty until details of the completed review are available.

Give the link to the published review.
