# Supplementary material for: A systematic review and meta-analyses on initiation, adherence and outcomes of antiretroviral therapy in incarcerated people
Source: PLoS One. 2020 May 18;15(5):e0233355. doi: 10.1371/journal.pone.0233355 (PMC7233580; doi:10.1371/journal.pone.0233355)
Supplement: S2 File — Effective public health practice project (EPHPP) quality assessment tool for quantitative Studies. (PDF) [file pone.0233355.s005.pdf]

QUALITY ASSESSMENT TOOL FOR QUANTITATIVE STUDIES

EPHPP
Effective Public Health Practice Project

COMPONENT RATINGS

A) SELECTION BIAS

(Q1) Are the individuals selected to participate in the study likely to be representative of the target population?

1 Very likely
2 Somewhat likely
3 Not likely
4 Can't tell

(Q2) What percentage of selected individuals agreed to participate?

1 80 - 100% agreement
2 60 – 79% agreement
3 less than 60% agreement
4 Not applicable
5 Can't tell

RATE THIS SECTION	STRONG	MODERATE	WEAK
See dictionary	1	2	3

B) STUDY DESIGN

Indicate the study design

1 Randomized controlled trial
2 Controlled clinical trial
3 Cohort analytic (two group pre + post)
4 Case-control
5 Cohort (one group pre + post (before and after))
6 Interrupted time series
7 Other specify ______________________________
8 Can't tell

Was the study described as randomized? If NO, go to Component C.

No Yes

If Yes, was the method of randomization described? (See dictionary)

No Yes

If Yes, was the method appropriate? (See dictionary)

No Yes

RATE THIS SECTION	STRONG	MODERATE	WEAK
**See dictionary**	1	2	3

C) CONFOUNDERS

(Q1) Were there important differences between groups prior to the intervention?

1 Yes
2 No
3 Can't tell

The following are examples of confounders:

1 Race
2 Sex
3 Marital status/family
4 Age
5 SES (income or class)
6 Education
7 Health status
8 Pre-intervention score on outcome measure

(Q2) If yes, indicate the percentage of relevant confounders that were controlled (either in the design (e.g. stratification, matching) or analysis)?

1 80 – 100% (most)
2 60 – 79% (some)
3 Less than 60% (few or none)
4 Can't Tell

RATE THIS SECTION	STRONG	MODERATE	WEAK
See dictionary	1	2	3

D) BLINDING

(Q1) Was (were) the outcome assessor(s) aware of the intervention or exposure status of participants?

1 Yes
2 No
3 Can't tell

(Q2) Were the study participants aware of the research question?

1 Yes
2 No
3 Can't tell

RATE THIS SECTION	STRONG	MODERATE	WEAK
See dictionary	1	2	3

E) DATA COLLECTION METHODS

(Q1) Were data collection tools shown to be valid?

1 Yes
2 No
3 Can't tell

(Q2) Were data collection tools shown to be reliable?

1 Yes
2 No
3 Can't tell

RATE THIS SECTION	STRONG	MODERATE	WEAK
See dictionary	1	2	3

F) WITHDRAWALS AND DROP-OUTS

(Q1) Were withdrawals and drop-outs reported in terms of numbers and/or reasons per group?

1 Yes
2 No
3 Can't tell
4 Not Applicable (i.e. one time surveys or interviews)

(Q2) Indicate the percentage of participants completing the study. (If the percentage differs by groups, record the lowest).

1 80 -100%
2 60 - 79%
3 less than 60%
4 Can't tell
5 Not Applicable (i.e. Retrospective case-control)

RATE THIS SECTION	STRONG	MODERATE	WEAK	
**See dictionary**	1	2	3	Not Applicable

G) INTERVENTION INTEGRITY

(Q1) What percentage of participants received the allocated intervention or exposure of interest?

1 80 -100%
2 60 - 79%
3 less than 60%
4 Can't tell

(Q2) Was the consistency of the intervention measured?

1 Yes
2 No
3 Can't tell

(Q3) Is it likely that subjects received an unintended intervention (contamination or co-intervention) that may influence the results?

4 Yes
5 No
6 Can't tell

H) ANALYSES

(Q1) Indicate the unit of allocation (circle one)

community organization/institution practice/office individual

(Q2) Indicate the unit of analysis (circle one)

community organization/institution practice/office individual

(Q3) Are the statistical methods appropriate for the study design?

1 Yes
2 No
3 Can't tell

(Q4) Is the analysis performed by intervention allocation status (i.e. intention to treat) rather than the actual intervention received?

1 Yes
2 No
3 Can't tell

GLOBAL RATING

COMPONENT RATINGS

Please transcribe the information from the gray boxes on pages 1-4 onto this page. See dictionary on how to rate this section.

**A**	**SELECTION BIAS**	**STRONG**	**MODERATE**	**WEAK**	
		1	2	3	
**B**	**STUDY DESIGN**	**STRONG**	**MODERATE**	**WEAK**	
		1	2	3	
**C**	**CONFOUNDERS**	**STRONG**	**MODERATE**	**WEAK**	
		1	2	3	
**D**	**BLINDING**	**STRONG**	**MODERATE**	**WEAK**	
		1	2	3	
**E**	**DATA COLLECTION METHOD**	**STRONG**	**MODERATE**	**WEAK**	
		1	2	3	
**F**	**WITHDRAWALS AND DROPOUTS**	**STRONG**	**MODERATE**	**WEAK**	
		1	2	3	Not Applicable

GLOBAL RATING FOR THIS PAPER (circle one):

1	STRONG	(no WEAK ratings)
2	MODERATE	(one WEAK rating)
3	WEAK	(two or more WEAK ratings)

With both reviewers discussing the ratings:

Is there a discrepancy between the two reviewers with respect to the component (A-F) ratings?

No Yes

If yes, indicate the reason for the discrepancy

1 Oversight
2 Differences in interpretation of criteria
3 Differences in interpretation of study

Final decision of both reviewers (circle one):

1 STRONG
2 MODERATE
3 WEAK
