# Supplementary material for: A systematic review and meta-analyses on initiation, adherence and outcomes of antiretroviral therapy in incarcerated people
Source: PLoS One. 2020 May 18;15(5):e0233355. doi: 10.1371/journal.pone.0233355 (PMC7233580; doi:10.1371/journal.pone.0233355)
Supplement: Attachment — Submitted filename: Response to Reviewers.docx [file pone.0233355.s007.docx]

**Journal Requirements:**

1. *Please update the methods section by explaining that no relevant studies were detected post Oct. 2018 (with the date of the last alert)*

**Response:** The methods section has been updated to explain that no relevant studies were detected post Oct 2018 with the last alert received on 28 March 2020 (p. 7 line 148).

**Response to editor’s questions:**

1. *Single screening is less efficient than double screening. It influences the number of studies missed. Please at least add a limitation in the discussion section. Waffenschmidt, S., Knelangen, M., Sieben, W., Bühn, S., & Pieper, D. (2019). Single screening versus conventional double screening for study selection in systematic reviews: a methodological systematic review. BMC medical research methodology, 19(1), 132.*

**Response:** a limitation has been added to indicate that there could be missed studies as screening was performed by a single reviewer (p. 42 line 611).

2. *Fig 1. Please add n for the last box on the right (“absence of other studies measuring the outcome/exposure).*

**Response:** number of studies to which there were no other studies measuring the same outcome/exposure has been specified.
